# 11-(*p*-Tolylsulfonyl)-8,14,24-trioxa-11,22,23-triazatetracyclo[19.2.1.0^2,7^.0^15,20^]tetracosa-1(23),2,4,6,15,17,19,21-octaene

**DOI:** 10.1107/S1600536809025070

**Published:** 2009-07-08

**Authors:** Xia Tian, Jian-Rong Han, Xiao-Li Zhen, Zhen-Chao Li, Shou-Xin Liu

**Affiliations:** a College of Sciences, Hebei University of Science & Technology, Shijiazhuang 050018, People’s Republic of China; b College of Chemical & Pharmaceutical Engineering, Hebei University of Science & Technology, Shijiazhuang 050018, People’s Republic of China

## Abstract

In the title compound, C_25_H_23_N_3_O_5_S, the central 1,3,4-oxadiazole ring makes dihedral angles of 35.05 (7), 23.68 (7) and 82.55 (8)°, with the three benzene rings. In the crystal structure, the packing is stabilized by weak non-classical inter­molecular C—H⋯O hydrogen bonds, which link the mol­ecules into an infinite network.

## Related literature

For related structures, see: Du, Hua & Jin (2001 ▶). For applications and synthesis of fluorescent sensors, see: Tong *et al.* (2000 ▶); Silva *et al.* (2000 ▶); Valeur & Leray (2000 ▶). For reference geometrical data, see: Du, Hua, Wang & Yan (2001 ▶).
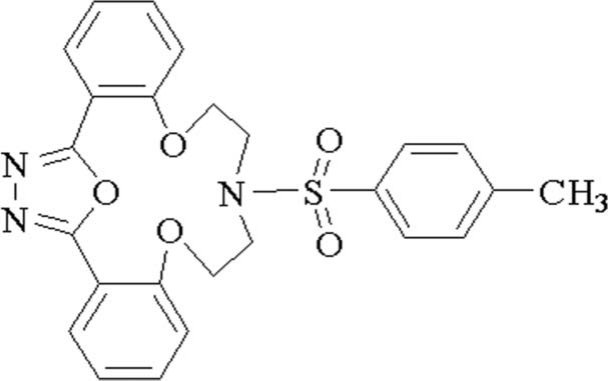


## Experimental

### 

#### Crystal data

C_25_H_23_N_3_O_5_S
*M* *_r_* = 477.52
Monoclinic, 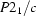

*a* = 13.0474 (19) Å
*b* = 9.6809 (14) Å
*c* = 18.261 (3) Å
β = 97.479 (3)°
*V* = 2286.9 (6) Å^3^
*Z* = 4
Mo *K*α radiation
μ = 0.19 mm^−1^
*T* = 294 K
0.30 × 0.22 × 0.20 mm

#### Data collection

Bruker SMART CCD area-detector diffractometer
Absorption correction: multi-scan (*SADABS*; Sheldrick, 1996 ▶) *T*_min_ = 0.932, *T*_max_ = 0.964
12599 measured reflections
4656 independent reflections
2878 reflections with *I* > 2σ(*I*)
*R*_int_ = 0.044

#### Refinement

*R*[*F* ^2^ > 2σ(*F* ^2^)] = 0.041
*wR*(*F* ^2^) = 0.106
*S* = 1.01
4656 reflections
308 parameters
H-atom parameters constrained
Δρ_max_ = 0.20 e Å^−3^
Δρ_min_ = −0.33 e Å^−3^

### 

Data collection: *SMART* (Bruker, 1999 ▶); cell refinement: *SAINT* (Bruker, 1999 ▶); data reduction: *SAINT*; program(s) used to solve structure: *SHELXS97* (Sheldrick, 2008 ▶); program(s) used to refine structure: *SHELXL97* (Sheldrick, 2008 ▶); molecular graphics: *SHELXTL* (Sheldrick, 2008 ▶); software used to prepare material for publication: *SHELXTL*.

## Supplementary Material

Crystal structure: contains datablocks I, global. DOI: 10.1107/S1600536809025070/pv2175sup1.cif

Structure factors: contains datablocks I. DOI: 10.1107/S1600536809025070/pv2175Isup2.hkl

Additional supplementary materials:  crystallographic information; 3D view; checkCIF report

## Figures and Tables

**Table 1:** Hydrogen-bond geometry (Å, °)

*D*—H⋯*A*	*D*—H	H⋯*A*	*D*⋯*A*	*D*—H⋯*A*
C10—H10*A*⋯O4^i^	0.97	2.52	3.424 (3)	154
C20—H20⋯O4^i^	0.93	2.39	3.314 (3)	173
C5—H5⋯O4^ii^	0.93	2.51	3.253 (3)	137

Symmetry codes: (i) 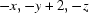; (ii) 
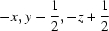
.

**Fig. 1.**
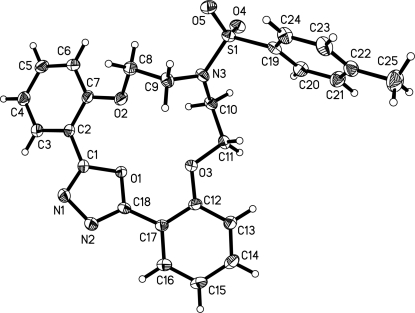
The molecular structure of (I) with displacement ellipsoids for non-H atoms drawn at the 30% probability level.

**Fig. 2.**
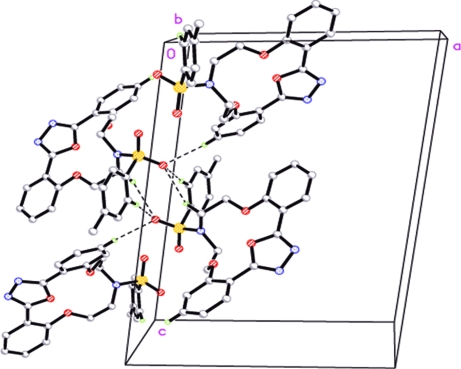
Packing diagram for (I), with H bonds drawn as dashed lines; H-atoms not involved in interactions have been excluded.

## supplementary crystallographic information

### Comment

The development of fluorescent sensors for organic molecules is of great practical importance in chemical, biological, and pharmaceutical sciences (Silva *et al.*, 2000; Valeur *et al.*, 2000). Therefore, the design and synthesis of new functionalized macrocycles for selective recognition of other species is of great interest to chemists. Many functionalized macrocycles have been synthesized and employed to develop fluorescent sensors (Tong *et al.*, 2000). 2,5-Diaryl-1,3,4-oxadizole forms chiral macrocyclic phosphoramidate receptors with phosphorus oxychloride, and their crystal structures have been reported (Du, Hua & Jin, 2001). As part of an investigation of the potential recognition properties of macrocycles, we now reported the synthesis and structure of the title compound, (I).

The molecular structure of (I) is presented in Fig. 1. The oxadizole ring (O1/C1/N1/N2/C18) is nearly planar, with an r.m.s. deviation for fitted atoms of 0.0017 Å. It makes dihedral angles of 35.05 (7), 23.68 (7) and 82.55 (8)°, respectively, with the benzene rings (C2—C7), (C12—C17) and (C19—C24). The crystal packing is stabilized by weak non-classical intermolecular C—H···O hydrogen bonds which link the molecules into an infinite network. The bond lengths and angles in (I) are within their normal ranges (Du, Hua, Wang & Yan 2001).

### Experimental

2,5-Di(*o*-hydroxyphenyl)-1,3,4-oxadiazole (0.8 g, 3.0 mmol), K_2_CO_3_ (1.4 g, 10 mmol) and tri-(*p*-phenylsulfonyl) diethanol amine (2.3 g, 4 mmol) were added and dissolved in 50 ml of DMF and the mixture was stirred at 413 K for 20 h giving a colourless precipitate. The product was isolated, recrystallized from ethyl acetate then dried in a vacuum to give the pure compound in 65% yield. colourless single crystals of (I) suitable for X-ray analysis were obtained by slow evaporation of ethyl acetate.

### Refinement

The H atoms were included in calculated positions (C—H = 0.93–0.97 Å) and refined as riding with *U*iso(H) = 1.2*U*eq(C)or 1.5*U*eq(methyl C).

### Crystal data

**Table:**

C_25_H_23_N_3_O_5_S	*F*(000) = 1000
*M**_r_* = 477.52	*D*_x_ = 1.387 Mg m^−^^3^
Monoclinic, *P*2_1_/*c*	Mo *K*α radiation, λ = 0.71073 Å
Hall symbol: -P 2ybc	Cell parameters from 3043 reflections
*a* = 13.0474 (19) Å	θ = 2.4–24.5°
*b* = 9.6809 (14) Å	µ = 0.19 mm^−^^1^
*c* = 18.261 (3) Å	*T* = 294 K
β = 97.479 (3)°	Prism, colourless
*V* = 2286.9 (6) Å^3^	0.30 × 0.22 × 0.20 mm
*Z* = 4	

### Data collection

**Table:**

Bruker SMART CCD area-detector diffractometer	4656 independent reflections
Radiation source: fine-focus sealed tube	2878 reflections with *I* > 2σ(*I*)
graphite	*R*_int_ = 0.044
φ and ω scans	θ_max_ = 26.4°, θ_min_ = 1.6°
Absorption correction: multi-scan (*SADABS*; Sheldrick, 1996)	*h* = −14→16
*T*_min_ = 0.932, *T*_max_ = 0.964	*k* = −12→11
12599 measured reflections	*l* = −18→22

### Refinement

**Table:**

Refinement on *F*^2^	Primary atom site location: structure-invariant direct methods
Least-squares matrix: full	Secondary atom site location: difference Fourier map
*R*[*F*^2^ > 2σ(*F*^2^)] = 0.041	Hydrogen site location: inferred from neighbouring sites
*wR*(*F*^2^) = 0.106	H-atom parameters constrained
*S* = 1.00	*w* = 1/[σ^2^(*F*_o_^2^) + (0.0446*P*)^2^ + 0.4604*P*] where *P* = (*F*_o_^2^ + 2*F*_c_^2^)/3
4656 reflections	(Δ/σ)_max_ = 0.001
308 parameters	Δρ_max_ = 0.20 e Å^−^^3^
0 restraints	Δρ_min_ = −0.33 e Å^−^^3^

### Special details

**Table:**

Geometry. All e.s.d.'s (except the e.s.d. in the dihedral angle between two l.s. planes) are estimated using the full covariance matrix. The cell e.s.d.'s are taken into account individually in the estimation of e.s.d.'s in distances, angles and torsion angles; correlations between e.s.d.'s in cell parameters are only used when they are defined by crystal symmetry. An approximate (isotropic) treatment of cell e.s.d.'s is used for estimating e.s.d.'s involving l.s. planes.
Refinement. Refinement of *F*^2^ against ALL reflections. The weighted *R*-factor *wR* and goodness of fit *S* are based on *F*^2^, conventional *R*-factors *R* are based on *F*, with *F* set to zero for negative *F*^2^. The threshold expression of *F*^2^ > σ(*F*^2^) is used only for calculating *R*-factors(gt) *etc*. and is not relevant to the choice of reflections for refinement. *R*-factors based on *F*^2^ are statistically about twice as large as those based on *F*, and *R*- factors based on ALL data will be even larger.

### Fractional atomic coordinates and isotropic or equivalent isotropic displacement parameters (Å^2^)

**Table:**

	*x*	*y*	*z*	*U*_iso_*/*U*_eq_	
S1	0.08777 (4)	1.10726 (6)	0.12913 (3)	0.03359 (16)	
N1	0.52489 (14)	0.5209 (2)	0.19897 (11)	0.0429 (5)	
N2	0.56917 (14)	0.5766 (2)	0.13898 (11)	0.0433 (5)	
N3	0.18176 (14)	0.99856 (19)	0.12592 (10)	0.0368 (5)	
O1	0.40350 (11)	0.62864 (15)	0.12555 (8)	0.0346 (4)	
O2	0.26584 (14)	0.73372 (16)	0.20414 (9)	0.0478 (5)	
O3	0.35451 (12)	0.84722 (17)	0.04510 (8)	0.0452 (4)	
O4	−0.00564 (11)	1.04490 (16)	0.09383 (9)	0.0417 (4)	
O5	0.09239 (13)	1.15346 (17)	0.20368 (9)	0.0470 (4)	
C1	0.42829 (17)	0.5545 (2)	0.18849 (12)	0.0317 (5)	
C2	0.34817 (16)	0.5205 (2)	0.23424 (11)	0.0317 (5)	
C3	0.35206 (18)	0.3940 (2)	0.27068 (12)	0.0397 (6)	
H3	0.4059	0.3331	0.2658	0.048*	
C4	0.27714 (19)	0.3576 (2)	0.31399 (13)	0.0437 (6)	
H4	0.2803	0.2728	0.3381	0.052*	
C5	0.19776 (19)	0.4482 (3)	0.32114 (13)	0.0443 (6)	
H5	0.1467	0.4236	0.3498	0.053*	
C6	0.19281 (19)	0.5752 (2)	0.28641 (13)	0.0424 (6)	
H6	0.1393	0.6360	0.2924	0.051*	
C7	0.26729 (18)	0.6122 (2)	0.24268 (12)	0.0354 (5)	
C8	0.2383 (2)	0.8580 (2)	0.23870 (13)	0.0436 (6)	
H8A	0.2783	0.8672	0.2871	0.052*	
H8B	0.1656	0.8563	0.2448	0.052*	
C9	0.26014 (17)	0.9778 (2)	0.19023 (12)	0.0380 (6)	
H9A	0.2653	1.0614	0.2197	0.046*	
H9B	0.3266	0.9629	0.1731	0.046*	
C10	0.18136 (17)	0.9098 (2)	0.06069 (12)	0.0354 (5)	
H10A	0.1165	0.9236	0.0291	0.042*	
H10B	0.1832	0.8143	0.0769	0.042*	
C11	0.26861 (17)	0.9320 (2)	0.01498 (13)	0.0393 (6)	
H11A	0.2465	0.9067	−0.0360	0.047*	
H11B	0.2888	1.0285	0.0165	0.047*	
C12	0.42882 (17)	0.8148 (2)	0.00178 (12)	0.0351 (5)	
C13	0.43787 (18)	0.8787 (3)	−0.06523 (13)	0.0429 (6)	
H13	0.3909	0.9467	−0.0833	0.052*	
C14	0.51633 (19)	0.8415 (3)	−0.10495 (14)	0.0473 (7)	
H14	0.5211	0.8837	−0.1501	0.057*	
C15	0.58800 (19)	0.7424 (3)	−0.07867 (14)	0.0470 (7)	
H15	0.6413	0.7190	−0.1055	0.056*	
C16	0.57964 (18)	0.6787 (2)	−0.01234 (13)	0.0413 (6)	
H16	0.6278	0.6119	0.0054	0.050*	
C17	0.50033 (16)	0.7125 (2)	0.02867 (12)	0.0323 (5)	
C18	0.49498 (16)	0.6389 (2)	0.09783 (12)	0.0320 (5)	
C19	0.11113 (16)	1.2493 (2)	0.07299 (12)	0.0329 (5)	
C20	0.09314 (19)	1.2366 (2)	−0.00310 (13)	0.0418 (6)	
H20	0.0687	1.1537	−0.0244	0.050*	
C21	0.1115 (2)	1.3470 (3)	−0.04719 (14)	0.0483 (7)	
H21	0.0993	1.3378	−0.0983	0.058*	
C22	0.14769 (19)	1.4715 (2)	−0.01674 (15)	0.0450 (6)	
C23	0.16432 (19)	1.4821 (2)	0.05905 (15)	0.0491 (7)	
H23	0.1883	1.5651	0.0804	0.059*	
C24	0.14638 (18)	1.3728 (2)	0.10435 (14)	0.0425 (6)	
H24	0.1580	1.3825	0.1554	0.051*	
C25	0.1681 (3)	1.5912 (3)	−0.06588 (18)	0.0734 (9)	
H25A	0.1336	1.6721	−0.0511	0.110*	
H25B	0.1425	1.5692	−0.1162	0.110*	
H25C	0.2411	1.6082	−0.0616	0.110*	

### Atomic displacement parameters (Å^2^)

**Table:**

	*U*^11^	*U*^22^	*U*^33^	*U*^12^	*U*^13^	*U*^23^
S1	0.0316 (3)	0.0310 (3)	0.0389 (3)	0.0016 (2)	0.0070 (2)	−0.0041 (3)
N1	0.0330 (12)	0.0535 (13)	0.0413 (12)	0.0021 (10)	0.0018 (9)	0.0105 (10)
N2	0.0297 (11)	0.0533 (13)	0.0470 (12)	0.0021 (9)	0.0046 (9)	0.0092 (11)
N3	0.0369 (11)	0.0369 (11)	0.0345 (11)	0.0112 (9)	−0.0032 (8)	−0.0079 (9)
O1	0.0283 (8)	0.0425 (9)	0.0338 (9)	0.0033 (7)	0.0068 (7)	0.0083 (7)
O2	0.0724 (12)	0.0332 (9)	0.0422 (10)	0.0133 (8)	0.0241 (9)	0.0074 (8)
O3	0.0427 (10)	0.0574 (11)	0.0379 (9)	0.0199 (8)	0.0143 (8)	0.0123 (8)
O4	0.0284 (8)	0.0413 (9)	0.0558 (10)	−0.0063 (7)	0.0069 (7)	−0.0041 (8)
O5	0.0565 (11)	0.0475 (10)	0.0388 (10)	0.0057 (8)	0.0130 (8)	−0.0086 (8)
C1	0.0338 (13)	0.0307 (12)	0.0296 (12)	−0.0003 (10)	0.0005 (10)	0.0015 (10)
C2	0.0346 (13)	0.0328 (12)	0.0268 (12)	−0.0005 (10)	0.0011 (10)	0.0009 (10)
C3	0.0420 (14)	0.0397 (13)	0.0370 (14)	0.0039 (11)	0.0042 (11)	0.0031 (12)
C4	0.0531 (16)	0.0376 (13)	0.0414 (14)	−0.0006 (12)	0.0099 (12)	0.0082 (12)
C5	0.0472 (15)	0.0469 (15)	0.0415 (14)	−0.0025 (12)	0.0163 (12)	0.0051 (12)
C6	0.0444 (14)	0.0436 (15)	0.0415 (14)	0.0086 (11)	0.0144 (12)	0.0017 (12)
C7	0.0447 (14)	0.0359 (13)	0.0261 (12)	0.0028 (11)	0.0065 (10)	0.0017 (10)
C8	0.0556 (16)	0.0386 (14)	0.0376 (14)	0.0100 (12)	0.0100 (12)	−0.0022 (11)
C9	0.0374 (13)	0.0351 (13)	0.0394 (14)	0.0019 (10)	−0.0033 (11)	−0.0012 (11)
C10	0.0348 (13)	0.0323 (12)	0.0382 (13)	0.0060 (10)	0.0013 (10)	−0.0053 (11)
C11	0.0410 (14)	0.0378 (13)	0.0382 (13)	0.0092 (11)	0.0019 (11)	0.0056 (11)
C12	0.0318 (13)	0.0421 (14)	0.0323 (13)	0.0005 (11)	0.0079 (10)	−0.0021 (11)
C13	0.0377 (14)	0.0531 (16)	0.0382 (14)	0.0018 (12)	0.0060 (11)	0.0086 (12)
C14	0.0455 (16)	0.0631 (17)	0.0344 (14)	−0.0098 (13)	0.0097 (12)	0.0043 (13)
C15	0.0371 (14)	0.0630 (17)	0.0436 (15)	−0.0064 (13)	0.0159 (12)	−0.0067 (14)
C16	0.0343 (14)	0.0472 (15)	0.0436 (15)	0.0004 (11)	0.0098 (11)	−0.0036 (12)
C17	0.0282 (12)	0.0350 (12)	0.0339 (13)	−0.0025 (10)	0.0054 (10)	−0.0024 (10)
C18	0.0254 (12)	0.0338 (12)	0.0370 (13)	−0.0006 (10)	0.0044 (10)	−0.0028 (10)
C19	0.0266 (12)	0.0301 (12)	0.0415 (14)	0.0044 (9)	0.0022 (10)	−0.0040 (11)
C20	0.0510 (15)	0.0307 (13)	0.0437 (15)	−0.0045 (11)	0.0056 (12)	−0.0087 (12)
C21	0.0591 (17)	0.0454 (15)	0.0414 (15)	−0.0025 (13)	0.0096 (13)	−0.0019 (13)
C22	0.0445 (15)	0.0355 (14)	0.0558 (17)	0.0005 (11)	0.0093 (12)	0.0040 (13)
C23	0.0549 (17)	0.0289 (13)	0.0609 (18)	−0.0068 (12)	−0.0022 (13)	−0.0063 (13)
C24	0.0474 (15)	0.0337 (14)	0.0441 (15)	−0.0008 (11)	−0.0022 (12)	−0.0059 (12)
C25	0.094 (2)	0.0480 (17)	0.080 (2)	−0.0076 (16)	0.0186 (19)	0.0143 (16)

### Geometric parameters (Å, °)

**Table:**

S1—O5	1.4268 (16)	C10—C11	1.512 (3)
S1—O4	1.4355 (16)	C10—H10A	0.9700
S1—N3	1.6227 (18)	C10—H10B	0.9700
S1—C19	1.765 (2)	C11—H11A	0.9700
N1—C1	1.292 (3)	C11—H11B	0.9700
N1—N2	1.410 (2)	C12—C13	1.390 (3)
N2—C18	1.295 (3)	C12—C17	1.405 (3)
N3—C9	1.467 (3)	C13—C14	1.377 (3)
N3—C10	1.468 (3)	C13—H13	0.9300
O1—C1	1.358 (2)	C14—C15	1.381 (3)
O1—C18	1.359 (2)	C14—H14	0.9300
O2—C7	1.370 (3)	C15—C16	1.376 (3)
O2—C8	1.426 (3)	C15—H15	0.9300
O3—C12	1.365 (2)	C16—C17	1.393 (3)
O3—C11	1.439 (3)	C16—H16	0.9300
C1—C2	1.458 (3)	C17—C18	1.459 (3)
C2—C3	1.392 (3)	C19—C24	1.379 (3)
C2—C7	1.403 (3)	C19—C20	1.384 (3)
C3—C4	1.381 (3)	C20—C21	1.378 (3)
C3—H3	0.9300	C20—H20	0.9300
C4—C5	1.376 (3)	C21—C22	1.384 (3)
C4—H4	0.9300	C21—H21	0.9300
C5—C6	1.381 (3)	C22—C23	1.377 (3)
C5—H5	0.9300	C22—C25	1.510 (3)
C6—C7	1.383 (3)	C23—C24	1.381 (3)
C6—H6	0.9300	C23—H23	0.9300
C8—C9	1.508 (3)	C24—H24	0.9300
C8—H8A	0.9700	C25—H25A	0.9600
C8—H8B	0.9700	C25—H25B	0.9600
C9—H9A	0.9700	C25—H25C	0.9600
C9—H9B	0.9700		
			
O5—S1—O4	119.22 (10)	H10A—C10—H10B	107.4
O5—S1—N3	107.43 (10)	O3—C11—C10	108.23 (17)
O4—S1—N3	108.21 (9)	O3—C11—H11A	110.1
O5—S1—C19	108.71 (10)	C10—C11—H11A	110.1
O4—S1—C19	105.52 (10)	O3—C11—H11B	110.1
N3—S1—C19	107.21 (10)	C10—C11—H11B	110.1
C1—N1—N2	106.20 (18)	H11A—C11—H11B	108.4
C18—N2—N1	106.15 (17)	O3—C12—C13	123.8 (2)
C9—N3—C10	119.84 (17)	O3—C12—C17	116.69 (19)
C9—N3—S1	120.75 (15)	C13—C12—C17	119.5 (2)
C10—N3—S1	119.07 (14)	C14—C13—C12	120.1 (2)
C1—O1—C18	103.30 (16)	C14—C13—H13	120.0
C7—O2—C8	118.99 (17)	C12—C13—H13	120.0
C12—O3—C11	119.08 (17)	C13—C14—C15	121.0 (2)
N1—C1—O1	112.27 (18)	C13—C14—H14	119.5
N1—C1—C2	128.1 (2)	C15—C14—H14	119.5
O1—C1—C2	119.65 (18)	C16—C15—C14	119.3 (2)
C3—C2—C7	119.0 (2)	C16—C15—H15	120.3
C3—C2—C1	119.2 (2)	C14—C15—H15	120.3
C7—C2—C1	121.7 (2)	C15—C16—C17	121.2 (2)
C4—C3—C2	120.9 (2)	C15—C16—H16	119.4
C4—C3—H3	119.5	C17—C16—H16	119.4
C2—C3—H3	119.5	C16—C17—C12	118.9 (2)
C5—C4—C3	119.3 (2)	C16—C17—C18	118.3 (2)
C5—C4—H4	120.4	C12—C17—C18	122.82 (19)
C3—C4—H4	120.4	N2—C18—O1	112.08 (19)
C4—C5—C6	121.0 (2)	N2—C18—C17	127.8 (2)
C4—C5—H5	119.5	O1—C18—C17	120.08 (19)
C6—C5—H5	119.5	C24—C19—C20	119.8 (2)
C5—C6—C7	120.1 (2)	C24—C19—S1	120.53 (18)
C5—C6—H6	120.0	C20—C19—S1	119.62 (17)
C7—C6—H6	120.0	C21—C20—C19	119.9 (2)
O2—C7—C6	123.7 (2)	C21—C20—H20	120.1
O2—C7—C2	116.57 (19)	C19—C20—H20	120.1
C6—C7—C2	119.7 (2)	C20—C21—C22	121.1 (2)
O2—C8—C9	108.12 (18)	C20—C21—H21	119.4
O2—C8—H8A	110.1	C22—C21—H21	119.4
C9—C8—H8A	110.1	C23—C22—C21	118.0 (2)
O2—C8—H8B	110.1	C23—C22—C25	121.6 (2)
C9—C8—H8B	110.1	C21—C22—C25	120.4 (2)
H8A—C8—H8B	108.4	C22—C23—C24	121.9 (2)
N3—C9—C8	114.2 (2)	C22—C23—H23	119.1
N3—C9—H9A	108.7	C24—C23—H23	119.1
C8—C9—H9A	108.7	C19—C24—C23	119.3 (2)
N3—C9—H9B	108.7	C19—C24—H24	120.4
C8—C9—H9B	108.7	C23—C24—H24	120.4
H9A—C9—H9B	107.6	C22—C25—H25A	109.5
N3—C10—C11	116.19 (19)	C22—C25—H25B	109.5
N3—C10—H10A	108.2	H25A—C25—H25B	109.5
C11—C10—H10A	108.2	C22—C25—H25C	109.5
N3—C10—H10B	108.2	H25A—C25—H25C	109.5
C11—C10—H10B	108.2	H25B—C25—H25C	109.5
			
C1—N1—N2—C18	0.1 (2)	C11—O3—C12—C17	−168.81 (19)
O5—S1—N3—C9	−7.8 (2)	O3—C12—C13—C14	178.8 (2)
O4—S1—N3—C9	−137.76 (17)	C17—C12—C13—C14	0.2 (3)
C19—S1—N3—C9	108.86 (18)	C12—C13—C14—C15	−1.1 (4)
O5—S1—N3—C10	165.50 (16)	C13—C14—C15—C16	1.0 (4)
O4—S1—N3—C10	35.56 (19)	C14—C15—C16—C17	−0.1 (4)
C19—S1—N3—C10	−77.82 (18)	C15—C16—C17—C12	−0.8 (3)
N2—N1—C1—O1	0.2 (2)	C15—C16—C17—C18	178.9 (2)
N2—N1—C1—C2	179.1 (2)	O3—C12—C17—C16	−178.0 (2)
C18—O1—C1—N1	−0.4 (2)	C13—C12—C17—C16	0.8 (3)
C18—O1—C1—C2	−179.42 (19)	O3—C12—C17—C18	2.3 (3)
N1—C1—C2—C3	−34.2 (3)	C13—C12—C17—C18	−178.9 (2)
O1—C1—C2—C3	144.6 (2)	N1—N2—C18—O1	−0.4 (2)
N1—C1—C2—C7	145.6 (2)	N1—N2—C18—C17	−179.5 (2)
O1—C1—C2—C7	−35.6 (3)	C1—O1—C18—N2	0.5 (2)
C7—C2—C3—C4	0.7 (3)	C1—O1—C18—C17	179.69 (19)
C1—C2—C3—C4	−179.5 (2)	C16—C17—C18—N2	23.2 (3)
C2—C3—C4—C5	−0.1 (4)	C12—C17—C18—N2	−157.1 (2)
C3—C4—C5—C6	−0.8 (4)	C16—C17—C18—O1	−155.8 (2)
C4—C5—C6—C7	1.1 (4)	C12—C17—C18—O1	23.8 (3)
C8—O2—C7—C6	41.7 (3)	O5—S1—C19—C24	10.7 (2)
C8—O2—C7—C2	−140.3 (2)	O4—S1—C19—C24	139.72 (18)
C5—C6—C7—O2	177.4 (2)	N3—S1—C19—C24	−105.10 (19)
C5—C6—C7—C2	−0.5 (3)	O5—S1—C19—C20	−168.80 (18)
C3—C2—C7—O2	−178.5 (2)	O4—S1—C19—C20	−39.8 (2)
C1—C2—C7—O2	1.7 (3)	N3—S1—C19—C20	75.4 (2)
C3—C2—C7—C6	−0.4 (3)	C24—C19—C20—C21	0.6 (3)
C1—C2—C7—C6	179.8 (2)	S1—C19—C20—C21	−179.84 (19)
C7—O2—C8—C9	170.55 (19)	C19—C20—C21—C22	−0.1 (4)
C10—N3—C9—C8	−78.4 (3)	C20—C21—C22—C23	−0.4 (4)
S1—N3—C9—C8	94.9 (2)	C20—C21—C22—C25	179.6 (2)
O2—C8—C9—N3	78.3 (2)	C21—C22—C23—C24	0.3 (4)
C9—N3—C10—C11	−70.9 (3)	C25—C22—C23—C24	−179.6 (2)
S1—N3—C10—C11	115.71 (19)	C20—C19—C24—C23	−0.7 (3)
C12—O3—C11—C10	159.53 (19)	S1—C19—C24—C23	179.78 (18)
N3—C10—C11—O3	87.7 (2)	C22—C23—C24—C19	0.2 (4)
C11—O3—C12—C13	12.5 (3)		

### Hydrogen-bond geometry (Å, °)

**Table:**

*D*—H···*A*	*D*—H	H···*A*	*D*···*A*	*D*—H···*A*
C10—H10A···O4^i^	0.97	2.52	3.424 (3)	154
C20—H20···O4^i^	0.93	2.39	3.314 (3)	173
C5—H5···O4^ii^	0.93	2.51	3.253 (3)	137

Symmetry codes: (i) −*x*, −*y*+2, −*z*; (ii) −*x*, *y*−1/2, −*z*+1/2.
